# Perfluorooctanesulfonic Acid–Induced Toxicity on Zebrafish Embryos in the Presence or Absence of the Chorion

**DOI:** 10.1002/etc.4899

**Published:** 2020-12-14

**Authors:** J. Erik Mylroie, Mitchell S. Wilbanks, Ashley N. Kimble, Kimberly T. To, Catherine S. Cox, Sheila J. McLeod, Kurt A. Gust, David W. Moore, Edward J. Perkins, Natàlia Garcia‐Reyero

**Affiliations:** ^1^ Bennet Aerospace Raleigh North Carolina USA; ^2^ Environmental Laboratory, US Army Engineer Research & Development Center Vicksburg Mississippi; ^3^ Oak Ridge Institute for Science and Education, Environmental Laboratory, US Army Engineer Research & Development Center Vicksburg Mississippi

**Keywords:** Zebrafish embryo, Perfluorooctanesulfonic acid, Chorion, Peroxisome proliferator–activated receptor, Perfluoroalkyl and polyfluoroalkyl substances

## Abstract

Perfluorooctanesulfonic acid (PFOS) is a perfluorinated compound used in many industrial and consumer products. It has been linked to a broad range of adverse effects in several species, including zebrafish (*Danio rerio*). The zebrafish embryo is a widely used vertebrate model to elucidate potential adverse effects of chemicals because it is amenable to medium and high throughput. However, there is limited research on the full extent of the impact the chorion has on those effects. Results from the present study indicate that the presence of the chorion affected the timing and incidence of mortality as well as morphometric endpoints such as spinal curvature and swim bladder inflation in zebrafish embryos exposed to PFOS. Furthermore, removal of the chorion prior to exposure resulted in a lower threshold of sensitivity to PFOS for effects on transcriptional expression within the peroxisome proliferator–activated receptor (PPAR) nuclear signaling pathway. Perturbation of PPAR pathway gene expression can result in disruption of metabolic signaling and regulation, which can adversely affect development, energy availability, and survival. It can be concluded that removal of the chorion has significant effects on the timing and incidence of impacts associated with PFOS exposure, and more research is warranted to fully elucidate the protective role of the chorion and the critical timing of these events. *Environ Toxicol Chem* 2021;40:780–791. Published 2020. This article is a US Government work and is in the public domain in the USA. *Environmental Toxicology and Chemistry* published by Wiley Periodicals LLC on behalf of SETAC.

## INTRODUCTION

Perfluoroalkyl and polyfluoroalkyl substances (PFAS) are a group of chemicals that have been widely used in numerous applications including waterproofing, fire retardants, firefighting foams, surfactants, and agrochemicals for over 50 yr (Armitage et al. 2006). Perfluorooctanesulfonic acid (PFOS), an 8–carbon chain PFAS, is fully substituted with fluorine atoms, making it an extremely stable molecule and resistant to environmental degradation (Wang et al. 2015; Liu et al. 2019). Contamination with PFOS in soil and water sources has been well documented throughout the world (Giesy and Kannan 2001; Ahrens and Bundschuh 2014; Wang et al. 2017; DeWitt et al. 2019). Compounding the problem of persistent, widespread contamination is that PFOS can bioaccumulate in individual organisms and biomagnify within the food chain (Martin et al. 2004; Haddad et al. 2017; Liu et al. 2019). Studies have shown that exposure to PFOS may cause a myriad of adverse effects in both aquatic and terrestrial organisms, including humans. These include several types of cancer as well as hepatotoxicity, immunotoxicity, neurotoxicity, delayed development, behavioral effects, reproductive toxicity, thyroid toxicity, and impacts on the microbiome (Lau et al. 2003; Thibodeaux et al. 2003; Wolf et al. 2007; Du et al. 2013; Chen et al. 2016; Negri et al. 2017; Lai et al. 2018; DeWitt et al. 2019; Sunderland et al. 2019). Because of their widespread contamination and impacts on animals and humans, various actions including the treatment and remediation of contaminated soil and water and the elimination and/or replacement of PFAS‐containing materials have been taken to manage and regulate PFAS, specifically PFOS, to help mitigate the current global contamination (National Ground Water Association 2017; Cordner et al. 2019).

Zebrafish (*Danio rerio*) is an established model used to understand the potential adverse effects of chemicals, stressors, and environmental pollutants. In particular, the zebrafish embryonic stage has been used extensively as a medium‐ and high‐throughput screening method where results can be applied to other vertebrates, including humans (Bambino and Chu 2017). Previous studies have examined the effects of PFOS exposure on zebrafish embryo development, and the morphological effects found in these studies included, but are not limited to, uninflated swim bladder, spinal curvature, pericardial edema, decreased length, and tissue necrosis (Shi et al. 2008; Huang et al. 2010; Hagenaars et al. 2011; Zhang et al. 2011; Zheng et al. 2012; Ulhaq et al. 2013a; Jantzen et al. 2016; Ortiz‐Villanueva 2018). Multiple studies have also shown that PFOS has effects on zebrafish embryo movement behavior as a result of both mechanical and potentially neurotoxic mechanisms (Huang et al. 2010; Ulhaq et al. 2013b; Hagenaars et al. 2014; Jantzen et al. 2016; Gaballah et al. 2020). The effects of PFOS on zebrafish embryo gene expression have also been investigated, and numerous genes and biological pathways associated with the response to PFOS have been identified (Shi et al. 2008, 2009; Shi and Zhou 2010; Du et al. 2013; Jantzen et al. 2016; Tse et al. 2016; Martínez et al. 2019a; Yao et al. 2019). Given the structural similarity to fatty acids, it is hypothesized that PFOS may disrupt a number of biological pathways, particularly those related to lipid metabolism (Zeng et al. 2019). One specific pathway identified as relevant to PFOS‐related toxicity is the peroxisome proliferator–activated receptor (PPAR) signaling pathway, which controls several important metabolic processes and is vital to zebrafish development and maintenance (Ibabe et al. 2005; Jantzen et al. 2016; Tse et al. 2016; Sant et al. 2018; Martínez et al. 2019b). There are 3 main classes of PPARs (PPARα, PPARγ, and PPARβ/δ), which are involved in metabolism, cell differentiation, tissue development, and inflammation (Varga et al. 2011; Bervejillo and Ferreira 2019). Specifically, PPARα activation results in fatty acid catabolism and energy production, PPARγ activation results in fatty acid synthesis as well as storage when energy sources are abundant, and PPARβ/δ plays a role in directing fatty acid catabolism in high energy–requiring tissues like cardiac muscle (Varga et al. 2011; Bervejillo and Ferreira 2019).

Although the zebrafish is considered a relevant model organism to understand toxicity, several sources of variability have been identified that can affect the outcomes reported by researchers (Hamm et al. 2019). One of the main methodological sources of variability is the retention of the chorion, the outermost membrane of the zebrafish egg, during exposure. The chorion is an acellular envelope pierced by pore canals and has been shown to block the movement of molecules >4 kDa in molecular weight (Pelka et al. 2017). The ability of a chemical to cross the chorion is affected not only by the size but also by physicochemical properties, ionic charge, and the use of a chemical “vehicle,” such as a solvent (Finn 2007; Kais et al. 2013; Kim and Tanguay 2014). During development, the structure and permeability of the chorion change, including a time‐dependent increase in permeability at 48 h postfertilization (hpf; Kais et al. 2013). The presence or absence of chorion could be important in understanding the potential hazard of chemicals. For instance, it has been shown that dechorination slightly improved concordance with mouse, rat, and rabbit teratogenicity tests (Ball et al. 2014). This would be consistent with studies showing that uptake of PFOS in zebrafish embryos is initially slow and then increases after 48 hpf (Huang et al. 2010; Vogs et al. 2019).

To better understand PFOS‐induced toxicity on zebrafish embryos, it is important to gain an understanding of how it will be impacted by the presence of the chorion. We tested whether the chorion had an effect on the timing and incidence of any of the adverse effects observed as a result of PFOS exposure. Zebrafish embryos were enzymatically dechorinated; then, both chorinated and dechorinated embryos were exposed to PFOS over the course of 120 hpf, and effects on development, morphology, behavior, and PPAR signaling pathway gene expression were examined.

## MATERIALS AND METHODS

### Chemicals

Heptadecafluorooctanesulfonic acid potassium salt (PFOS; Chemical Abstracts Service no. 2795‐39‐3, >98% purity, product no. 77282, lot no. BCBS9941) was obtained from Sigma‐Aldrich. A stock solution of 4 mg/L was made directly in estradiol (E2) media without methylene blue (Varga 2016) by magnetically stirring the solution overnight in the dark, and subsequent nominal concentrations of 3, 2, 1, and 0.6 mg/L were made in E2 media from the stock solution. The E2 media was used as the control solution for the exposure.

### Zebrafish husbandry and exposure

Wild‐type, AB strain adult zebrafish (Zebrafish International Research Center) were housed on ZebTEC racks (Tecniplast) in tanks filled with fish water (reverse osmosis water supplemented with ocean salts and sodium bicarbonate) and maintained at 28.5 °C with a 14:10‐h light:dark cycle. Water quality parameters including conductivity, salinity, and pH (see Supplemental Data, Table S1) were measured using a YSI Professional Plus multimeter, whereas alkalinity, total hardness, and nitrate were measured using a Lamotte Smart3 colorimeter. Water quality parameters were maintained at levels described by Varga (2016), and adult fish were fed GEMMA 500 μm fish food (Skretting). For breeding, adult fish (2:1 male to female ratio) were housed overnight in an iSpawn (Tecniplast) breeding chamber filled with fish water, with the same water quality parameters and housing parameters. Embryos were obtained on the morning of the exposure by spawning adults according to the manufacturer's recommendations (2:1 ratio of males to females, separating fish the day prior to spawning with female fish on top and spawning during the dawn light cycle). Once collected and counted, embryos that were to remain chorinated were washed and surface‐sanitized using a bleach solution as modified from a protocol from Varga and Murray (2016). Dechorination of embryos was achieved by pronase digestion (Sigma‐Aldrich; p8811, lot no. SLBS 8998) at approximately 4.5 hpf using a modified method from Truong et al. (2011) by conducting pronase digestion under gentle agitation for 7 min, followed by immediately rinsing embryos in E2 media for 7 min.

At approximately 7 hpf, approximately 200 viable embryos were exposed in Petri dishes containing the appropriate PFOS exposure solution or control media (E2 media), and then each embryo was placed in an individual well in 48‐well cell culture–treated plates containing 1 mL of the matching solution, where they underwent static exposure conditions for 5 d. Twenty‐two replicates were used for each treatment, where treatment = each concentration × chorion state (chorion state = chorion on or off). Each plate contained both chorinated and dechorinated embryos for one exposure concentration, yielding a total of 6 plates. Plates were covered with the lid, sealed with Parafilm® (Bemis), and placed in an incubator at 28.5 °C with a 14:10‐h light:dark cycle. Embryos were observed once daily at 24 and 48 hpf and then twice daily from 72 to 120 hpf. Mortality and any morphological/developmental abnormalities were recorded during these observations.

### Analytical chemistry

Samples of PFOS for analytical chemistry were collected immediately before and after the 120‐h exposure. Pre‐exposure samples were taken from the exposure solution before it was added to the 48‐well plates. Postexposure samples were taken by pooling all of the remaining exposure solution in the 44 wells of each concentration (pooled 22 each from chorinated and dechorinated) after the embryos had been removed from the wells at the end of the exposure (~120 hpf).

To quantify PFOS from both pre‐ and postexposure solutions, a volume of methanol equal to that of the sample was added in the original collection vessel. Once the MeOH was added, the samples were diluted as needed to fall within the instrument's linear range for PFOS. In the final dilution internal standard was present at 0.5 µg/L. The sample was in a solution of 50:50 (MeOH:H_2_O) for analysis by triple quadrupole liquid chromatography‐mass spectrometry (LC‐MS/MS). All samples were analyzed using an Agilent 1290 Infinity Binary Pump LC coupled to an Agilent 6495B triple quadrupole MS/MS with Jet Streaming Technology and electrospray ionization (ESI). Chromatographic separation was performed using a Water's Atlantis dC18 column (2.1 × 150 mm, 5 µm). An Agilent Eclipse Plus C18 RRHD column (3.0 × 50 mm, 1.8 µm) was used to delay any possible PFOS that is inherently in the system. Data acquisition was performed in dynamic multiple reaction monitoring mode using negative‐mode ESI. Chromatographic separation was achieved by gradient elution with a flow rate of 0.5 mL/min using 5 mM ammonium acetate in high performance liquid chromatography water as mobile phase A and MeOH as mobile phase B. The analytical column was held at a temperature of 50 °C during separation.

### Locomotor activity

At approximately 24 hpf, embryos were assessed for locomotor activity via spontaneous tail flicks per minute. Each live embryo was visualized under a stereo‐ or inverted microscope, and tail flicks were counted by hand over the course of 1 min. Four plates were assayed seperately but simultaneously by 4 different researchers to minimize the impact of time as a factor in this assay.

### Targeted gene expression

Total RNA was isolated from individual 120‐hpf embryos using Clontech NucleoSpin RNA XS kits (Takara Bio), following the manufacturer's recommendations. Four replicates, with a replicate being a single embryo, were chosen at random from each treament except for the embryos exposed to 4 mg/L PFOS (because of almost 100% mortality) for total RNA isolation. Criteria for acceptable RNA integrity included an RNA integrity number >8.0 from the Agilent 2200 TapeStation and a NanoDrop 2000 (ThermoFisher Scientific) 260/280 spectrophotometric reading ≥1.9.

Total RNA (100 ng input) from each replicate was used to synthesize cDNA using the RT2 First Strand Kit (Qiagen) before undergoing gene expression analysis using Custom Qiagen RT2 profiler polymerase chain reaction (PCR) arrays (catalog no. CAPZ14085). Seventy‐nine genes involved in PPAR signaling were used as targets, and 5 housekeeping genes were also included for use in normalization in this array (see Supplemental Data, Table S2). The raw cycle threshold (Ct) values were normalized based on the geometric mean of the 5 housekeeping genes. Profiler arrays were run on an Applied Biosystems QuantStudio 6 Flex instrument using recommended cycling parameters. Cycling parameters included 1 cycle for 10 min at 95 °C, 40 cycles of 15 s at 95 °C, followed by 1 min at 60 °C, and then a dissociation (melting) curve (95 °C for 15 s, 60 °C for 1 min, 95 °C for 15 s). Differentially expressed genes for each treatment condition were mapped onto the Kyoto Encyclopedia of Genes and Genomes (KEGG; Kanehisa and Goto 2000) PPAR signaling pathway.

### Statistical analyses

The median lethal concentration (LC50) was calculated based on mortality at 120 hpf for the dechorinated and chorinated embryos separately using the trimmed Spearman‐Karber method in ToxCalc software (Ver 5.0; Tidepool Scientific Software). The median effective concentration (EC50) was calculated based on spinal curvature and swim bladder malformations such as improper swim bladder inflation for dechorinated and chorinated embryos separately using the Quest Graphi™ EC50 Calculator (AAT Bioquest, n.d.). Analytically measured PFOS concentrations were used in the calculation of both LC50 and EC50 values.

To check for plate effects, 4 internal controls were placed on each plate. Tail flick counts from the internal controls were compared to the negative control responses to assess whether the internal control responses were consistent across plates. Responses were compared visually. Analyses of the apical endpoints were performed in R (Ver 3.6.2; R Core Development Team 2019). There were 4 binary morphological endpoints assayed: 24 hpf mortality, 120 hpf mortality, 120 hpf axis (tail/spinal curvature), and 120 hpf (improper swim bladder development and embryo orientation). “Embryo orientation” refers to the embryo in any other state than its normal righted position. We reported the embryo as laying on its back or side as a positive hit versus being in its normal righted position. Improper swim bladder development and embryo orientation were combined because the responses were identical. The embryos from the 4‐mg/L group were excluded in the analysis of swim bladder development/embryo orientation because of near complete mortality in both the chorinated and dechorinated treatments (Table 1).

**Table 1:** Sample sizes per treatment group used for statistical analysis

		Treatment group				
Dose	Chorion	24 hpf Survival	24 hpf Tail flicks	120 hpf Survival	120 hpf Spinal curvature	120 hpf Orientation/swim bladder^a^
Control	+	24	21	24	24	22
Control	–	24	21	24	24	21
0.6 ppm	+	22	20	22	22	22
0.6 ppm	–	22	22	22	22	21
1.0 ppm	+	22	19	22	22	18
1.0 ppm	–	22	21	22	22	21
2.0 ppm	+	22	21	22	22	19
2.0 ppm	–	22	21	22	22	13
3.0 ppm	+	22	21	22	22	7
3.0 ppm	–	22	21	22	22	8
4.0 ppm	+	22	21	22	22	2
4.0 ppm	–	22	21	22	22	1

^a^ Sample sizes in this group vary greatly because of the inability to asses swim bladder inflation/embryo orientation in dead embryos.

A one‐sided Fisher's exact test was used to compare whether the treatment group had a higher number of affected embryos for a given endpoint compared to controls. For the comparisons of chorinated versus dechorinated, the dechorinated group was considered the treatment group. Effect sizes were calculated using Cohen's H for proportions and are reported for any significant pairwise comparisons. Pairwise comparisons for tail flick counts were performed using the Mann‐Whitney U test. Effect sizes were calculated as the standardized U statistic and are reported for any significant pairwise comparisons. For the treatment versus control within chorion groups, a Bonferroni‐corrected *p* = 0.05/5 was used to account for multiple comparisons across the 5 doses. For comparisons of the chorinated and dechorinated groups, a Bonferroni‐corrected *p* = 0.05/6 was used to account for comparisons within the control group and 5 doses. Effect sizes were classified based on a general rule of thumb, where treatments with an effect size >0.2 were considered to have evidence of a meaningful effect.

For gene expression analysis, Ct values from quantitative reverse‐transcription PCR runs were uploaded to Qiagen's GeneGlobe Data Analysis Center. The 2^(∆∆Ct)^ method was utilized to determine fold changes, and the *p* values were calculated using a Student's *t* test of the replicate 2^(∆Ct)^ values for each gene in the control and treatment groups. For these analyses, chorinated treatments were compared to the chorinated control and the dechorinated treatments were compared to the dechorinated control, with a significance threshold of *p* ≤ 0.05 and fold change threshold of ±1.5 being used for all comparisons. The chorinated control was also compared to the dechorinated control to determine if there were any gene expression differences inherent to chorination state.

## RESULTS

### Analytical chemistry

Measurements of PFOS in both the pre‐ and postexposure solutions indicated that exposure concentrations were lower than the nominal values, possibly due to absorption to glassware during solution preparation. In all cases except for the nominal 4‐mg/L solution, the pre‐exposure solution had a higher concentration than the postexposure solutions (Table 2). All references to PFOS concentrations from this point forward will be shown as the measured concentration from the preexposure PFOS solutions.

**Table 2:** Nominal and measured perfluorooctanesulfonic acid concentrations

Nominal PFOS concentration (mg/L)	PFOS in exposure solutions (mg/L)		
	Pre‐exposure	Postexposure	% Change
Negative control (E2 media)	0	0	0
0.6	0.37	0.26	–30%
1	0.71	0.43	–39%
2	1.53	1.3	–15%
3	2.55	1.78	–31%
4	3.26	3.51	7%

PFOS = perfluorooctanesulfonic acid; E2 = estradiol.

### Exposure and morphological assessment

**Figure 1:**
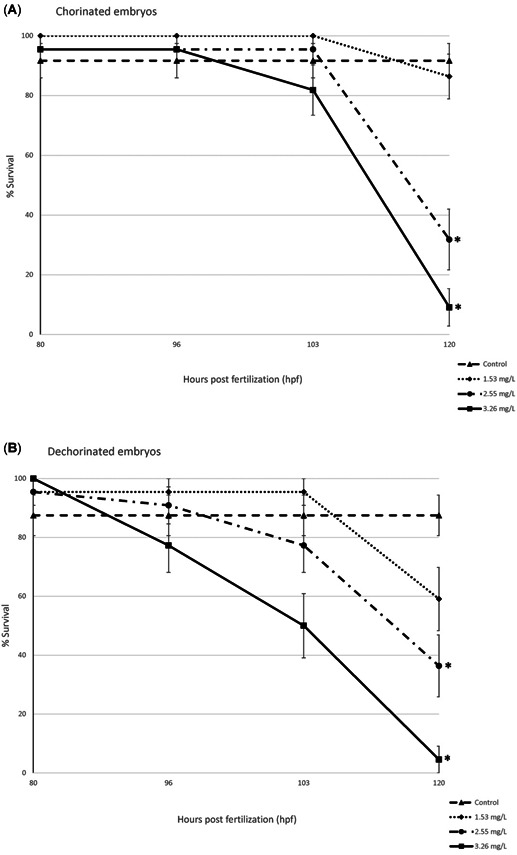
Survival over time for chorinated and dechorinated embryos (80–120 hpf). Error bars indicate standard error. 0.37 and 0.71 mg/L perfluorooctanesulfonic acid survival was not included because of no significant mortality over the time assayed. *Statistical significance using the Bonferroni‐corrected *p* value (*p* < 0.05).

Mortality due to PFOS exposure was first observed at approximately 96 hpf at the 3.26 mg/L concentration and occurred earlier in dechorinated embryos than chorinated embryos (Figure 1). All mortality in the other concentrations occurred after 96 hpf. At 120 hpf, both chorinated and dechorinated embryos exposed to either 2.55 or 3.26 mg/L PFOS had significantly higher mortality at 120 hpf (Figure 1; Supplemental Data, Figure S1). Comparison of the chorinated and dechorinated groups did not show evidence of group differences. There was no evidence of a difference in mortality in embryos exposed to PFOS at 0.71 or 0.37 mg/L PFOS regardless of the presence or absence of the chorion (Figure 1; Supplemental Data, Figure S1). The 120‐hpf LC50 for the dechorinated and chorinated embryos was 1.84 mg/L (95% confidence interval 0.83–4.06 mg/L) and 2.25 mg/L (95% confidence interval 1.39–3.62 mg/L), respectively.

**Figure 2:**
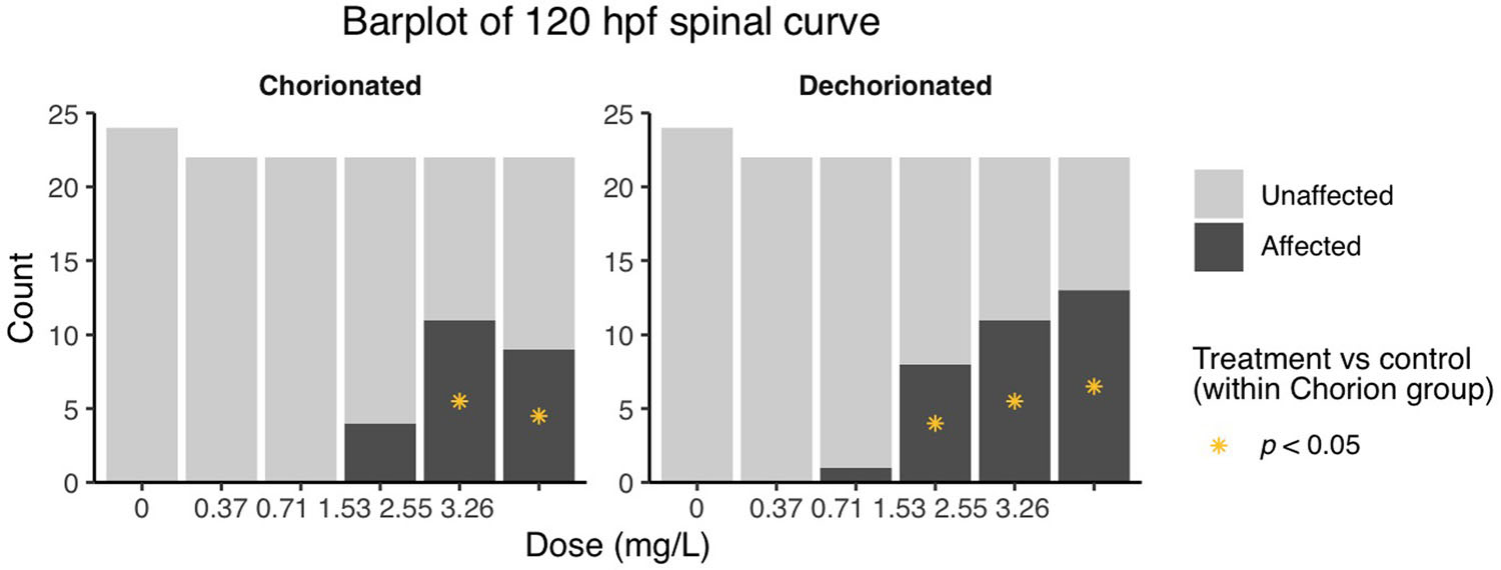
Abnormal spinal curvature by treatment group at 120 hpf. *Statistical significance using the Bonferroni‐corrected *p* value (*p* < 0.05).

Incidences of spine curvature in the dechorinated embryos increased with increasing PFOS concentrations (Figure 2), and the 1.53, 2.55, and 3.26 mg/L concentrations had significantly higher incidences of spine curvature compared to control. In the chorinated embryos, the 2.55 and 3.26 mg/L concentrations had significantly higher incidences of spine curvature. Differences among the chorinated and dechorinated embryos within the 2.55‐ and 3.26‐mg/L treatments were not statistically significant. At 120 hpf, the EC50 for spine curvature was 1.56 mg/L in the chorinated embryos and 1.37 mg/L in the dechorinated embryos.

**Figure 3:**
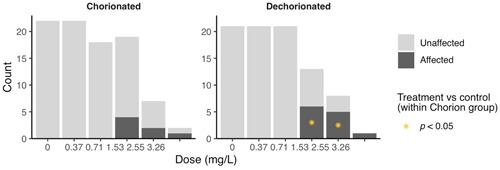
Uniflated swimbladder/abnormal orientation at 120 hpf. *Statistical significance using the Bonferroni‐corrected *p* value (*p* < 0.05).

When embryos were assessed for underinflated swim bladder or an on‐side orientation versus the normal righted embryo orientation at 120 hpf, the dechorinated embryos in the 1.53 and 2.55 mg/L PFOS exposure concentrations had statistically higher incidences relative to dechorinated controls. In contrast, the chorinated embryos in the 1.53 and 2.55 mg/L PFOS exposure concentrations were not significantly different from the chorinated controls (Figure 3). There were no significant differences among the chorinated and dechorinated groups within the 1.53 and 2.55 mg/L treatments. The EC50 for this morphological effect was 1.43 mg/L for the chorinated embryos and 1.36 mg/L for the dechorinated embryos.

### Locomotor activity

There was no evidence of differences between any treatment and their respective controls when assayed for 24‐hpf spontaneous tail flicks (Supplemental Data, Figure S2).

### Targeted transcript expression of the PPAR signaling network

**Figure 4:**
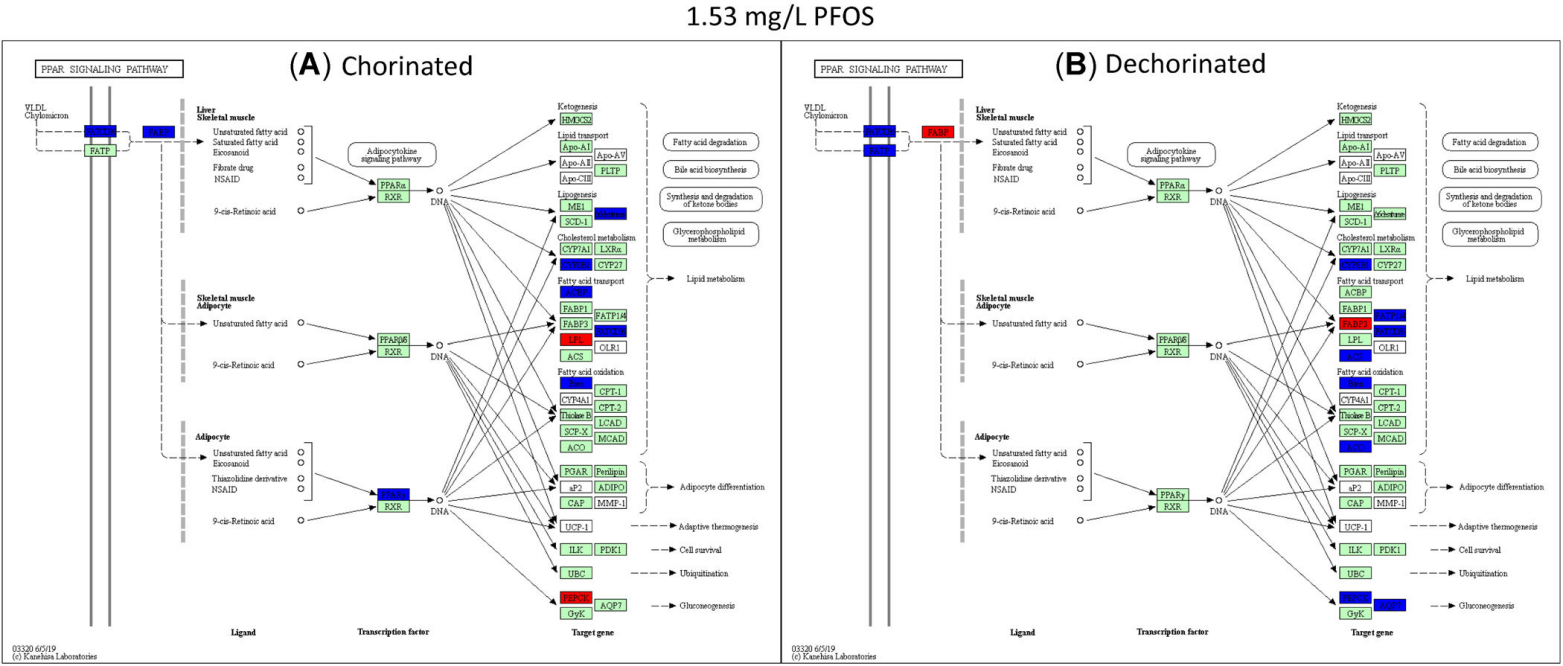
Kyoto Encyclopedia of Genes and Genomes pathway analysis showing the peroxisome proliferator–activated receptor pathway genes significantly expressed at 1.5‐fold and greater for the chorinated (**A**) and dechorinated (**B**) embryos exposed to 1.53 mg/L perfluorooctanesulfonic acid. Red indicates genes that were up‐regulated relative to the respective control, blue indicates genes that were down‐regulated relative to the respective control, and green indicates genes that were analyzed but did not differ from the respective control. PFOS = perfluorooctanesulfonic acid; PPAR = peroxisome proliferator–activated receptor.

**Figure 5:**
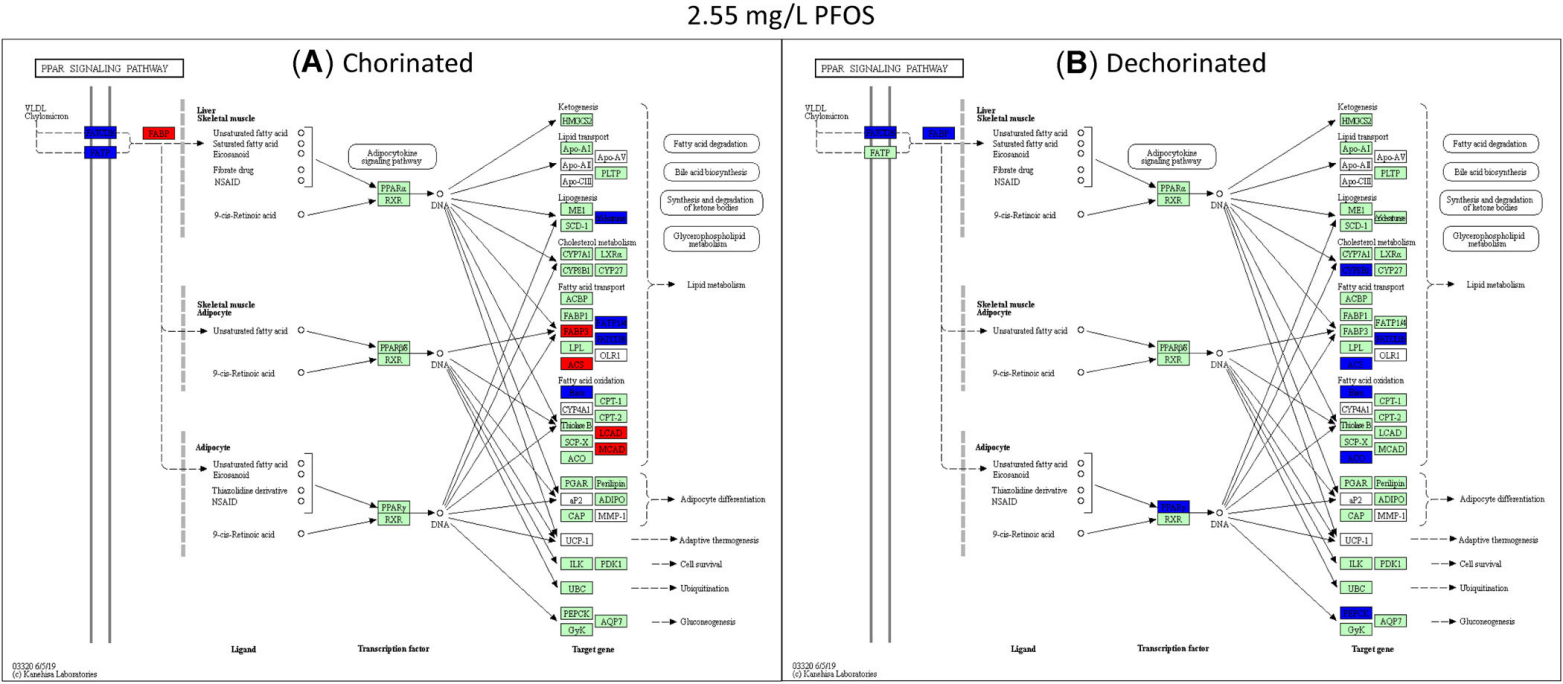
Kyoto Encyclopedia of Genes and Genomes pathway analysis showing the peroxisome proliferator–activated receptor pathway genes significantly expressed at 1.5‐fold and greater for the chorinated (**A**) and dechorinated (**B**) embryos exposed to 2.55 mg/L perfluorooctanesulfonic acid. Red indicates genes that were up‐regulated relative to the respective control, blue indicates genes that were down‐regulated relative to the respective control, and green indicates genes that were analyzed but did not differ from the respective control. PFOS = perfluorooctanesulfonic acid; PPAR = peroxisome proliferator–activated receptor.

Only one gene, lipoprotein lipase, was differentially expressed in the PPAR signaling network between the chorinated and dechorinated control embryos (Supplemental Data, Table S2). In contrast, numerous genes were differentially expressed relative to controls in both chorinated and dechorinated embryos as a result of PFOS exposure (Table 3). In the chorinated embryos, there were no significant differentially expressed transcripts at the 0.37 and 0.71 mg/L PFOS concentrations; however, the same concentrations showed multiple differentially expressed transcripts in the dechorinated embryos. In addition, the PFOS exposure elicited an increased number of differentially expressed transcripts in the dechorinated versus chorinated embryos, with totals of 27 and 24, respectively (Table 3). A survey of transcript expression comparing both chorination states indicated that several of the transcripts that were differentially expressed in response to the PFOS exposures were found in common between chorinated and dechorinated embryos (Figures 4 and 5). Further, the directional expression (increased vs decreased fold change) in response to PFOS was conserved for all differentially expressed transcripts found in common among chorinated and dechorinated embryos, as evidenced by no cases of gene transcripts being expressed in opposite directions (Table 3). There were, however, instances of gene transcripts having significant differential expression in response to PFOS in only one chorination state. For example, a total of 12 transcripts uniquely expressed in the chorinated embryos were almost all up‐regulated relative to the control, whereas dechorinated embryos displayed a total of 15 unique differentially expressed transcripts where 10 had decreased expression (Table 3; Supplemental Data, Figure S3).

**Table 3:** List of differentially expressed genes

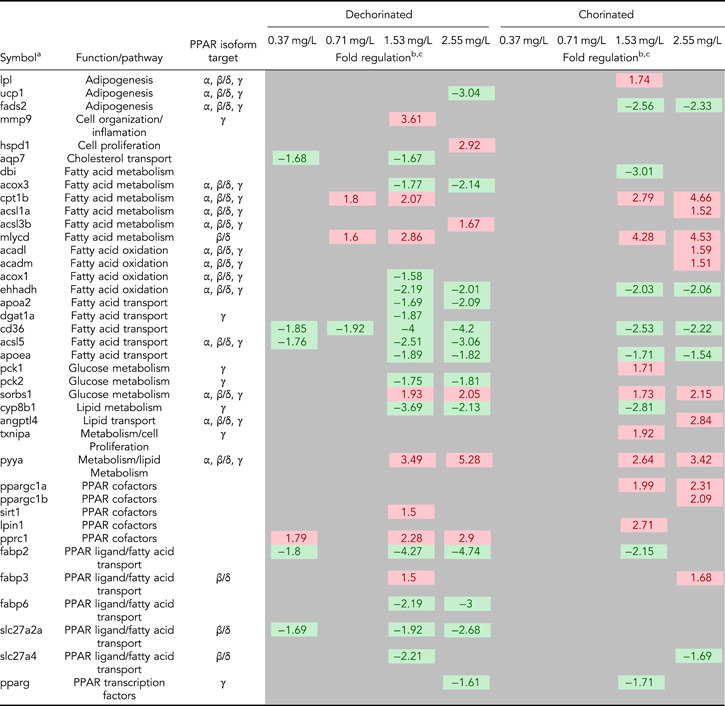										

^a^Gene symbols used are those found at the Zebrafish Information Network 2020. If no symbol was found, the closest orthologue found in GenBank (National Center for Biotechnology Information 2020) was used.

^b^Only genes with significance of *p* ≤ 0.05 and a fold change ≥ ±1.5 were considered significant.

^c^Fold changes colored green indicate genes down‐regulated when compared to their respective control, and fold changes colored red indicate genes up‐regulated when compared to their respective control.

PPAR = peroxisome proliferator–activated receptor.

The thrombospondin receptor/fatty acid transporter showed a significantly decreased expression at all doses in the dechorinated embryos and at the 2 highest PFOS exposures levels (1.53 and 2.55 mg/L) in the chorinated embryos (Table 3). The gene with the greatest magnitude of increased transcriptional expression in any treatment was peptide YYa (*pyya*), which had increased expression with increasing PFOS exposure concentrations (1.53 and 2.55 mg/L) in both chorination states (Table 3).

Transcripts of PPAR and associated coactivators showed differing patterns of expression with only one PPAR gene, *pparg* (PPARγ), differentially expressed in at least one concentration in both the chorinated (1.53 mg/L) and dechorinated (2.55 mg/L) embryos (–1.71‐ and –1.61‐fold, respectively). In all other PPAR‐associated coactivators, the differential expression was only significant in one chorination state. In the chorinated embryos, PPAR coactivators *pprgc1a* and *pprgc1b* were both up‐regulated in at least one PFOS concentration, with both being up‐regulated at the 2.55 mg/L concentration. For the dechorinated embryos, *pprc*, another PPAR coactivator, was up‐regulated at the 0.37, 1.53, and 2.55 mg/L concentrations (1.79‐, 2.28‐, and 2.9‐fold, respectively). A pathway‐level view of transcriptional expression (Figures 4 and 5) provides context for the PPAR signaling in response to PFOS exposures, where many of the trends are conserved across chorination states.

## DISCUSSION

Previous studies (Huang et al. 2010; Vogs et al. 2019) have indicated that the zebrafish chorion affects PFOS uptake before hatching because of limited permeability of PFOS through the chorion. The present study investigated whether the presence or absence of chorion had impacts on the incidence, severity, and/or timing of various morphological effects and targeted gene‐transcript expression by comparing chorinated and dechorinated embryos simultaneously exposed to PFOS from 7 to 120 hpf.

Our results indicated that removal of the chorion prior to PFOS exposure resulted in earlier onset of effects, increased sensitivity, and/or increased incidence of response. Although statistical analysis of mortality did not show a difference in chorinated versus dechorinated embryos within concentrations, mortality was observed earlier in the dechorinated embryos as opposed to the chorinated embryos (Figure 1). Moreover, the dechorinated embryos had a lower LC50 than the chorinated embryos (1.84 and 2.25 mg/L, respectively). Incidences of spinal curvature (example in Supplemental Data, Figure S4) and uninflated swim bladders relative to controls were increased in the dechorinated embryos (Figures 2 and 3). Earlier onset of morphometric malformations in response to PFOS exposures was also observed in dechorinated relative to chorinated embryos (Supplemental Data, Figure S5). Chorinated embryos did not exhibit malformations until after hatching, as observed in other studies (Huang et al. 2010; Hagenaars et al. 2011), providing more evidence that the chorion may act as a protective barrier against PFOS in early embryonic exposures.

Tail malformations due to PFOS exposure have been attributed to apoptosis (Shi et al. 2008; Huang et al. 2010) and muscle fiber alterations (Huang et al. 2010) or have been considered a secondary result of reduced cardiac output (Incardona et al. 2004). Even though changes in tail flicks were not observed in the present study, changes in tail morphology may explain why embryos are not able to fill their swim bladder with air. Thyroid disruption is another potential route leading to impaired swim bladder inflation (Stinckens et al. 2020). Although measuring effects on thyroid hormones was beyond the scope of the present study, other researchers have linked PFOS to thyroid disruption in zebrafish and other species (Chen et al. 2018).

Exposure to PFOS can disrupt energy metabolism, especially lipid metabolism, in zebrafish and other organisms, which can result in hepatic steatosis and other deleterious endpoints (Lee at al. 2019). It is thought that PFOS‐induced disruption of metabolism is caused, in part, through direct interactions with PPARs and thus affects the entire nuclear signaling pathway (Li et al. 2018; Lee et al. 2019; Yi et al. 2019). Consistent with this, several genes known to be regulated by PPAR signaling that are involved in lipid metabolism and gluconeogenesis were affected in both chorinated and dechorinated embryos (Table 3 and Figures 4 and 5; Supplemental Data, Figure S3). Because no transcripts were differentially expressed in the 0.37 and 0.71 mg/L exposures of the chorinated embryos but multiple transcripts were affected in the dechorinated embryos (Table 3), our data support the hypothesis that the chorion inhibits PFOS entry into the embryo, leading to a possibly delayed or decreased accumulation of PFOS.

Chorinated embryos had mixed responses when compared to dechorinated embryos relative to gene expression within a common pathway (PPAR signaling). In chorinated embryos, transcriptional expression for genes involved in fatty acid binding, lipogenesis, cholesterol metabolism, fatty acid transport, fatty acid oxidation, and gluconeogenesis were affected (overall 2 up/8 down at 1.53 mg/L PFOS; 5 up/6 down 2.55 mg/L PFOS; Figures 4 and 5). In dechorinated embryos, transcriptional expression for genes involved in fatty acid binding, lipogenesis, cholesterol metabolism, fatty acid transport, fatty acid oxidation, adaptive thermogenesis, and gluconeogenesis were affected (overall 2 up/10 down at 1.53 mg/L PFOS; 0 up/10 down 2.55 mg/L PFOS). Dechorinated embryos had more dysregulation of PPAR‐regulated genes and a higher proportion of decreased transcriptional expression than chorinated embryos. Notably, genes related to both lipid metabolism and gluconeogenesis were down‐regulated in dechorinated embryo exposures. Transcriptional responses consistent with a decrease in lipid metabolism and decreased expression of phosphoenolpyruvate carboxykinase 2 (*pck2*), which catalyzes the rate‐limiting reaction for gluconeogenesis, and aquaporin 7, important in glycerol transport in mammals (Mendez‐Gimenez et al. 2014), suggest a potential shift away from energy storage in favor of cellular energy production (Kramer 2016). Transcriptional expression of *pck1* was increased in chorinated embryos exposed to 1.53 mg/L PFOS but not in dechorinated embryos. Increased *ppargc1a* expression in chorinated embryos may contribute to increased fatty acid catabolism and gluconeogenesis, therefore contributing to increased availability of energy substrates (Lin et al. 2005).

In both chorinated and dechorinated embryos, malonyl‐CoA decarboxylase and carnitine palmitoyltransferase 1B (*cpt1b*) had increased transcriptional expression, which is consistent with a potential increase in fatty acid catabolism, particularly in the muscle. Forms of PPAR coactivators also had increased transcriptional expression in both chorinated (*ppargc1a* and *ppargc1b*) and dechorinated (*pprc1*) embryos, and increased expression of these coactivators has been shown to increase expression of genes involved in fatty acid catabolism in cellular assays including *cpt1* (Lin et al. 2003).

Peptide YY (PYY) is a signaling molecule that has been studied in depth in other animals, with an ever‐increasing focus on its role in zebrafish feeding behavior and development (Söderberg et al. 2000; Mathieu et al. 2005; Gonzalez and Unniappan 2010; Sundström et al. 2013; Velasco et al. 2018). It is part of a complex system that regulates appetite/feeding through signals sent to and from the central nervous system to peripheral organs like the gastrointestinal tract (Suzuki et al. 2010). Among other functions, it has been shown to inhibit gut motility and decrease appetite, leading to possible weight loss (Batterham et al. 2002; Boey et al. 2008; Shi et al. 2015). Furthermore, studies have shown that the PYY peptide can have effects on PPAR expression, and it is an integral part of the complex system that regulates metabolism (Nadal et al. 2017; Velasco et al. 2018). In the present study, *pyya* exhibited increased transcriptional expression in both chorinated and dechorinated embryos exposed to PFOS at the 1.53 and 2.55 mg/L exposure concentrations and had the highest magnitude of differential expression versus the control across all genes assayed (Table 3). Though not assayed in the present study, previous studies in zebrafish have shown that expression of neuropeptide Y (NPY) protein and the *npy* gene is decreased during PFOS exposure and that decreased expression is linked to an overall decrease in feeding behavior (Narnaware et al. 2000; Yokobori et al. 2012; Soengas et al. 2018; Opazo et al. 2019; Tu et al. 2019). This disruption in hunger/feeding signaling may play a role, along with other PFOS‐induced effects like disruption of the hypothalamic–pituitary–thyroid axis, in morphometric endpoints such as reduced fish length and weight that have been observed in other zebrafish PFOS exposures (Shi et al. 2009; Hagenaars et al. 2011, 2014; Cheng et al. 2016; Jantzen et al. 2016). Research has shown that PYY can increase energy expenditure using fat as an energy substrate, which may help to further explain the increased transcriptional expression of genes involved in fatty acid oxidation (Karra et al. 2009). However, it is important to note that PYY is involved in other aspects of development; and therefore, its increased expression because of PFOS exposure may be a result of and/or lead to other developmental problems like neural tube defect (Yuziriha et al. 2007; Chen et al. 2014). Through the complex interactions that control metabolism, it is plausible that PFOS mimics fatty acids in a way that interferes with critical signaling networks that control basic energy metabolism in the zebrafish by simultaneously binding with many molecules that regulate metabolism including the PPARs (Shabalina et al. 2016; Xu et al. 2016; Yi et al. 2019).

## CONCLUSIONS

Our data indicate that the chorion plays a significant role in the toxicity of PFOS to zebrafish embryos by decreasing sensitivity to PFOS, possibly by acting as a protective barrier to PFOS uptake into the embryo and thereby delaying potential adverse effects. Dechorinated embryos appeared to show greater sensitivity to PFOS exposure at the transcriptional and phenotypic levels. Taken in total, these results indicate that the presence of the chorion on embryos exposed to PFOS affects the severity and timing of both morphometric and transcriptional endpoints. Therefore, it is important that researchers take into consideration the potential role of the chorion in the uptake of certain chemicals when designing zebrafish embryo toxicity assays.

## Supplemental Data

The Supplemental Data are available on the Wiley Online Library at https://doi.org/10.1002/etc.4899.

## Disclaimer

The statements, opinions, or conclusions contained herein do not necessarily represent the statements, opinions, or conclusions of the Department of Defense or the US government.

## Supporting information

This article includes online‐only Supplemental Data.

Supporting information.

Supporting information.

Supporting information.

Supporting information.

Supporting information.

Supporting information.

Supporting information.

Supporting information.

## Data Availability

Data, associated metadata, and calculation tools are available from the corresponding author (Natalia.G.Vinas@erdc.dren.mil).
