# Supplementary figures and images for: Perfluorooctanesulfonic Acid–Induced Toxicity on Zebrafish Embryos in the Presence or Absence of the Chorion
Source: Environ Toxicol Chem. 2020 Dec 14;40(3):780–91. doi: 10.1002/etc.4899 (PMC7984204; doi:10.1002/etc.4899)

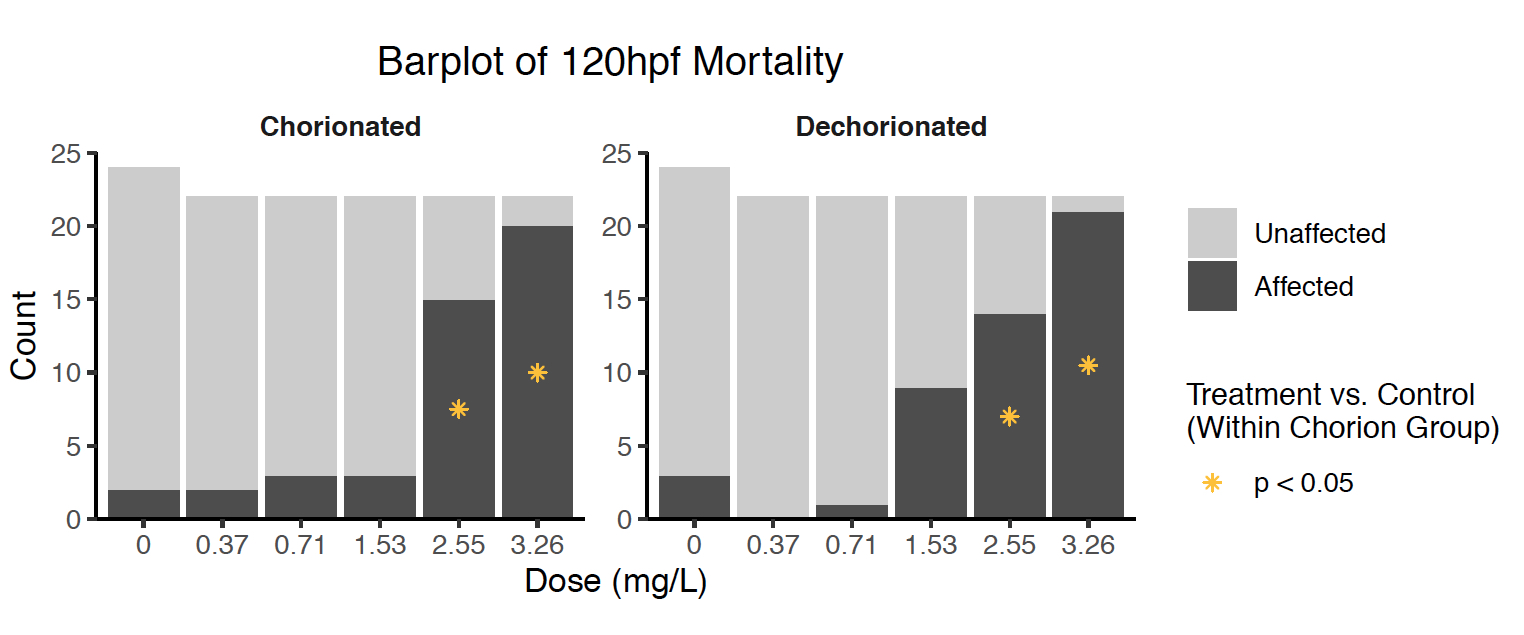

Supplement: Supplementary file 1 — Supporting information. [file ETC-40-780-s007.jpg]

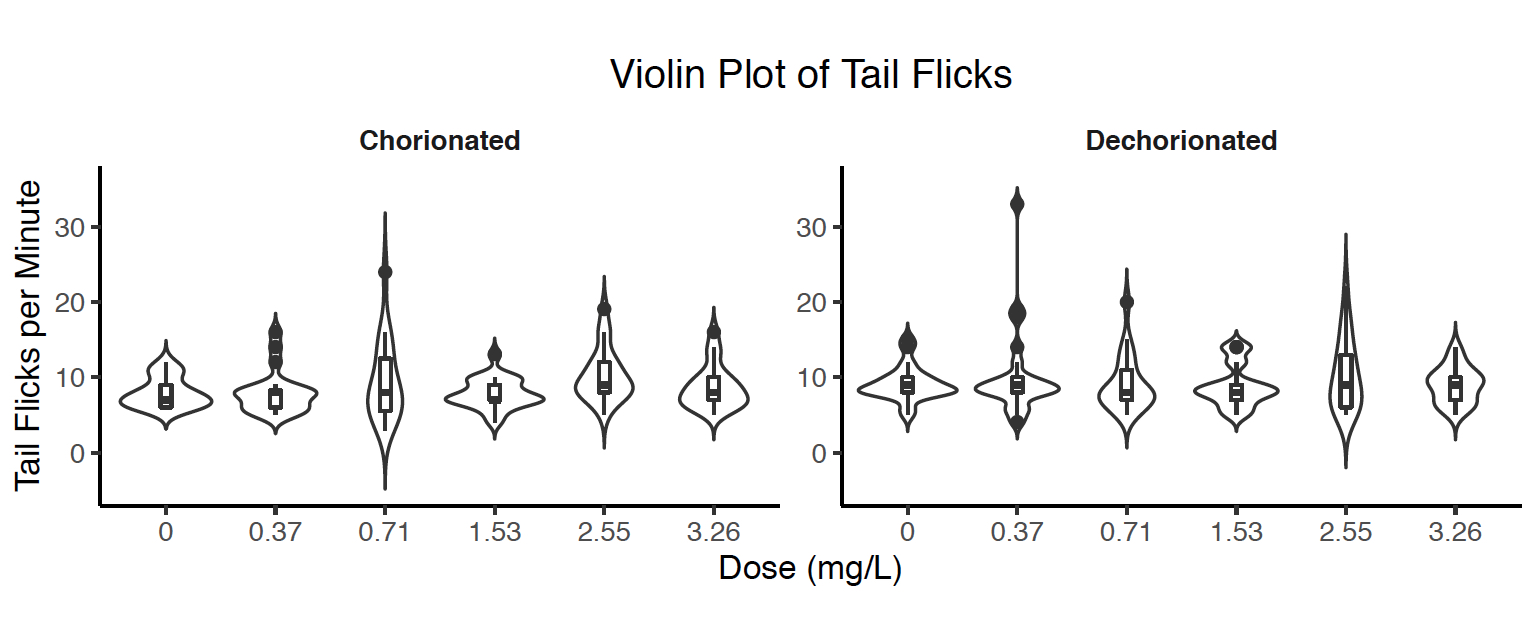

Supplement: Supplementary file 2 — Supporting information. [file ETC-40-780-s006.jpg]

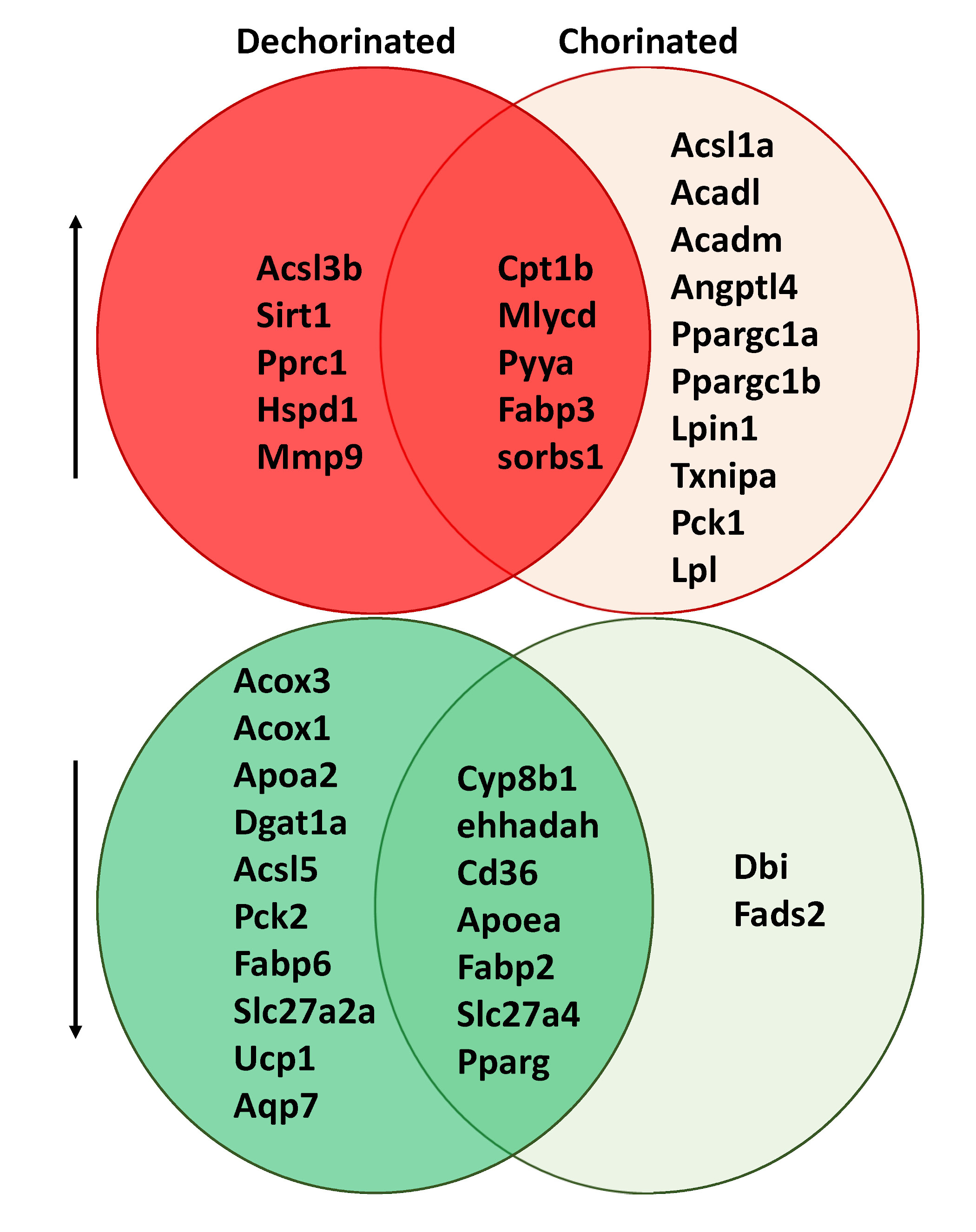

Supplement: Supplementary file 3 — Supporting information. [file ETC-40-780-s005.jpg]

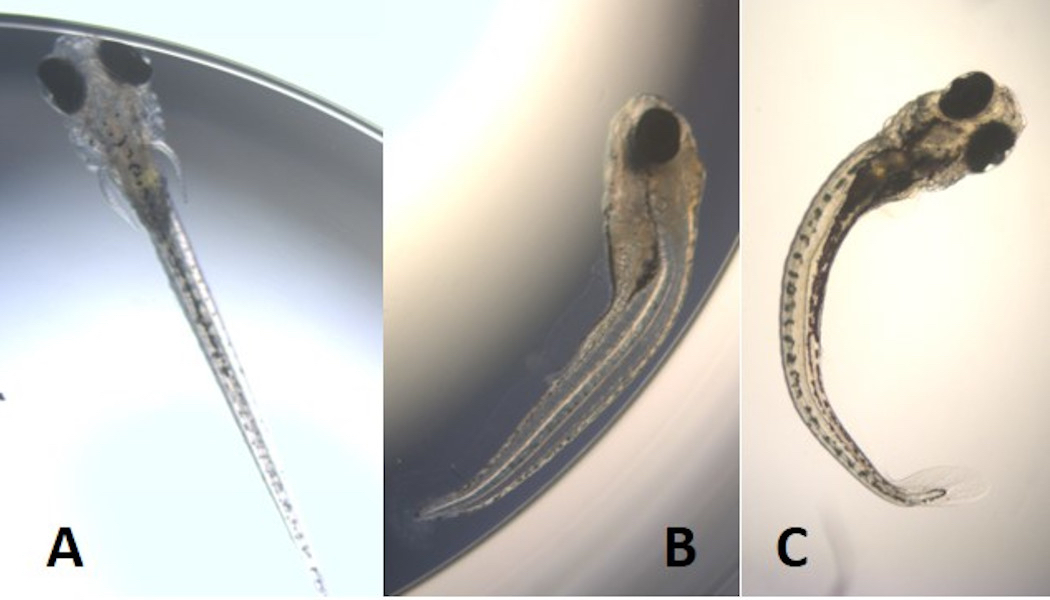

Supplement: Supplementary file 4 — Supporting information. [file ETC-40-780-s004.jpg]

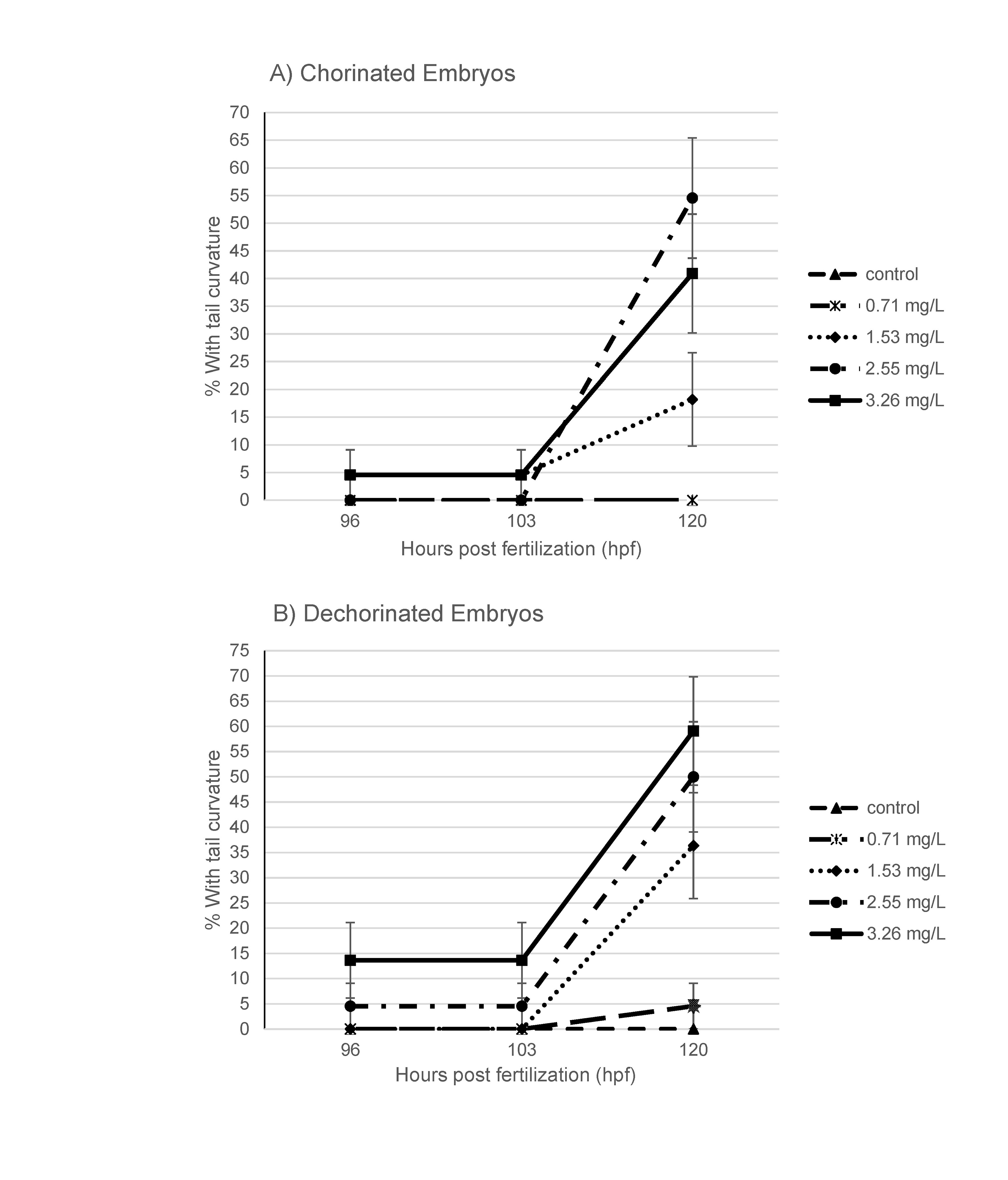

Supplement: Supplementary file 5 — Supporting information. [file ETC-40-780-s008.jpg]
