# Supplementary material for: Perfluorooctanesulfonic Acid–Induced Toxicity on Zebrafish Embryos in the Presence or Absence of the Chorion
Source: Environ Toxicol Chem. 2020 Dec 14;40(3):780–91. doi: 10.1002/etc.4899 (PMC7984204; doi:10.1002/etc.4899)
Supplement: Supplementary file 6 — Supporting information. [file ETC-40-780-s003.docx]

**Supplemental Table 1:** water quality parameters

| Exposure |  | | conductivity | salinity | pH | alkalinity | total hardness | NO_3_^-^ |
| --- | --- | --- | --- | --- | --- | --- | --- | --- |
|  |  | | uS/cm | ppt |  | ppm | ppm | ppm |
| E2 media | |  | 1120 | 0.59 | 7.63 | 40 | 109 | 4 |
| PFOS | mg/L | |  |  |  |  |  |  |
|  | 0.6 | | 1125 | 0.58 | 7.54 | 33 | 110 | 4 |
|  | 1 | | 1120 | 0.59 | 7.5 | 32 | 109 | 5 |
|  | 2 | | 1068 | 0.56 | 7.49 | 37 | 106 | 5 |
|  | 3 | | 1108 | 0.58 | 7.5 | 33 | 107 | 5 |
|  | 4 | | 1119 | 0.59 | 7.49 | 28 | 109 | 6 |
