# Supplementary material for: Perfluorooctanesulfonic Acid–Induced Toxicity on Zebrafish Embryos in the Presence or Absence of the Chorion
Source: Environ Toxicol Chem. 2020 Dec 14;40(3):780–91. doi: 10.1002/etc.4899 (PMC7984204; doi:10.1002/etc.4899)
Supplement: Supplementary file 8 — Supporting information. [file ETC-40-780-s001.docx]

|  | Dechorinated | | | | Chorinated | |
| --- | --- | --- | --- | --- | --- | --- |
| Canonical Pathway | 0.37 mg/L | 0.71 mg/L | 1.53 mg/L | 2.55 mg/L | 1.53 mg/L | 2.55 mg/L |
| LPS/IL-1 mediated inhibition of RXR function | 5.72E-04 |  | 5.70E-10 | 2.47E-07 |  | 6.12E-06 |
| Fatty acid activation | 2.29E-03 |  | 5.70E-05 | 3.16E-05 |  | 2.35E-05 |
| ϒ-linolenate biosynthesis II | 2.99E-03 |  | 9.92E-05 |  |  | 9.88E-08 |
| mitchochondrial L-carnitine shuttle pathway | 2.99E-03 |  |  |  |  | 4.08E-05 |
| LXR/RXR activation |  | 1.59E-02 |  |  | 3.21E-03 |  |
| fatty acid β-oxidation I |  |  | 2.84E-06 | 1.14E-06 |  | 2.29E-09 |
| FXR/RXR activation |  |  | 4.07E-06 |  | 8.83E-05 |  |
| type II diabetes mellitus signaling | 2.30E-04 | 1.86E-02 |  | 1.89E-06 |  |  |
| Maturity onset of diabetes of youn (MODY) signaling |  | 2.77E-03 |  |  |  |  |
| inhibition of angiogenesis by TSP1 |  | 4.48E-03 |  |  |  |  |
| atherosclerosis signaling |  | 1.65E-02 |  |  |  |  |
| adipogenesis pathway |  |  |  |  | 1.99E-06 |  |
| sirtuin signaling pathway |  |  |  |  | 1.03E-03 |  |
| fatty acid β-oxidation III (unsaturated, odd number) |  |  |  |  | 2.81E-03 |  |

**Supplemental Table 3**. Summary of top 5 canonical pathways each treatment (Qiagen Ingenuity Pathway Analysis)^a,b^.

^a^Blue color indicates top 5 canonical pathways found in both chorinated and dechorinated embryos. Green color indicates a top 5 canonical pathway found only in dechorinated embryo treatment. Gold color indicates top 5 canconical pathways only found in chorinated embryo treatment.

^b^QIAGEN Inc., https://www.qiagenbioinformatics.com/products/ingenuitypathway-analysis
